# Towards systemic and contextual priority setting for implementing the 2030 Agenda

**DOI:** 10.1007/s11625-017-0470-0

**Published:** 2017-09-12

**Authors:** Nina Weitz, Henrik Carlsen, Måns Nilsson, Kristian Skånberg

**Affiliations:** 1 Stockholm Environment Institute (SEI), P.O. Box 24218, 104 51 Stockholm, Sweden; 2 Department of Sustainable Development, Environmental Science and Engineering, KTH Royal Institute of Technology, 100 44 Stockholm, Sweden

**Keywords:** 2030 Agenda, Sustainable development goals (SDG), Systems analysis, Network analysis, Policy coherence

## Abstract

How the sustainable development goals (SDGs) interact with each other has emerged as a key question in the implementation of the 2030 Agenda, as it has potentially strong implications for prioritization of actions and their effectiveness. So far, analysis of interactions has been very basic, typically starting from one SDG, counting the number of interactions, and discussing synergies and trade-offs from the perspective of that issue area. This paper pushes the frontier of how interactions amongst SDG targets can be understood and taken into account in policy and planning. It presents an approach to assessing systemic and contextual interactions of SDG targets, using a typology for scoring interactions in a cross-impact matrix and using network analysis techniques to explore the data. By considering how a target interacts with another target and how that target in turn interacts with other targets, results provide a more robust basis for priority setting of SDG efforts. The analysis identifies which targets have the most and least positive influence on the network and thus guides, where efforts may be directed (and not); where strong positive and negative links sit, raising warning flags to areas requiring extra attention; and how targets that reinforce each others’ progress cluster, suggesting where important cross-sectoral collaboration between actors is merited. How interactions play out is context specific and the approach is tested on the case of Sweden to illustrate how priority setting, with the objective to enhance progress across all 17 SDGs, might change if systemic impacts are taken into consideration.

**Electronic supplementary material:**

The online version of this article (doi:10.1007/s11625-017-0470-0) contains supplementary material, which is available to authorized users.

## Introduction

In July 2016, 22 countries presented their first national voluntary reviews of the implementation of the sustainable development goals (SDGs) of the 2030 Agenda at the United Nation’s High-Level Political Forum (HLPF). The reviews revealed that, as countries now are moving to implementation, the complexity of the agenda is appearing in all its colours, and as a result, many countries appear to be somewhat at a loss when it comes to developing their action strategies in substantive terms: most reviews in 2016 focussed purely on procedure and institutional arrangements (UN 2016b).

Implementation is complicated by the fact that targets and goals interact and impact each other in different ways; in UN rhetoric, the agenda is “indivisible” and countries should implement the agenda as a whole (UN 2015). However, when it comes to action, governments and other actors have both competing priorities and limited budgets. Interests might clash and goals be seen to counteract each other. Most governments are not effectively organized to deal with multi-sectoral, multi-scale, multi-actor issues such as the SDGs. Furthermore, the knowledge base needed to address them in an informed way is insufficient (ICSU 2017). While the 17 SDGs and their associated 169 targets are fairly straightforward as individual goals (although they leave much room for national interpretation, see Weitz et al. 2015), the systemic properties of the system as a whole are poorly understood. How pursuing certain targets generate rippling effects by influencing other targets has been recognized as a critical knowledge gap in SDG implementation (UN 2016a). Yet, there have been relatively limited advances when it comes to practically filling this gap, despite the importance of these systemic impacts for the effectiveness of action and thus chances of maximizing progress on the SDGs overall.

The dynamics of how exactly targets interact with each other is an empirical question and the answer will be different in different contexts. It depends, for example, on the natural resource base (such as land or water availability), governance arrangements, what technologies are available, and political ideas of future pathways for sustainable development (Nilsson et al. 2016). Understanding interactions between targets requires quite detailed information, but it also requires the ability to maintain a holistic view of the system as a whole, since it is possible that one policy change can change the dynamics of the whole system. Research that disentangles interaction amongst the SDGs can support policy and decision makers seeking to ensure effective and coherent implementation across the governmental machinery.

In this paper, we take a first step towards analyzing SDG interactions with respect to its systemic and contextual character. It contributes to filling the research gap on the systemic properties of the SDGs by developing a transparent analytical approach that can support a whole-of-government perspective to SDG implementation in practice. Based on assessments of how SDG targets interact; how the achievement of one target may be inextricably linked to or create conditions for the achievement of another target, or alternatively may limit options, clash with or even make it impossible to reach another target, we show how targets most positively or negatively influenced by progress in other targets can be identified. We also show how clusters of highly interacting SDG targets can be identified, again based on assessment of how the targets interact. Highlighting how targets influence each other, and thus how the achievement of one target may depend on developments in other targets, we discuss implications for the prioritization of actions and collaboration amongst different government ministries with responsibility for the different policy areas covered by the SDGs.

It is important to stress that the approach developed relies on the scoring of interactions between each pair of SDG targets included in the analysis (and, of course, the mathematical machinery used to analyse these interaction scorings, as explained below). In this sense, the proposed methodology for analyzing systemic properties of the SDG targets is strictly bottom–up: all systemic properties identified, e.g., priorities of targets and cluster identifications, rely on the validity of these assessments, and any such assessment is going to be contentious. Different experts, different groups of stakeholders, and/or different groups of decision makers may reach different scoring results.

Our findings highlight how the realization of one ministry’s priority target hinges on how a target under the auspices of another ministry is pursued. Our hypothesis is that taking systemic effects into account provides a more robust understanding of these dynamics and alters which efforts should be prioritized in SDG implementation to enhance progress on the 2030 Agenda as a whole.

### A system perspective to the SDGs—the state of the art

Numerous efforts have been made to conceptualize and assess interaction amongst the SDGs. A literature overview^1^ suggests that there is a general call for further research and that the policy relevance of results has been limited (see, e.g., Hajer et al. 2015; Lu et al. 2015). Much research has taken off from a specific goal area and explored its links with other SDGs (see, e.g., Jha et al. 2016; Langlois et al. 2012; Collin and Casswell 2016). Alternatively, a subset of goals or targets such as how water, energy, and food target interact has been the focus of study (see, e.g., Yumkella and Yillia 2015; Ringler et al. 2013). Only a small number of research projects have focused on interaction across all goals (see, e.g., Nilsson et al. 2016; Boas et al. 2016; Le Blanc 2015). Le Blanc (2015) used network analysis techniques to establish the existence of links, basing their findings on interaction expressed in the wording of the SDGs. In further elaborations, Vladimirova and Le Blanc (2016) explored more links based on document review of UN reports, focusing on the case of education. Some quantitative methods for systems analysis have been applied to the SDGs, including cumulative effects assessment, multi-criteria decision analysis, and scenario analysis (see, e.g., Fleskens et al. 2013; Jayaraman et al. 2015; Jones 2016). Empirical research seeking to explain how interactions actually play out and what their policy implications are has been limited.

A focus on policy coherence and mainstreaming is evident in the SDG policy debate and literature (see, e.g., Nordbeck and Steurer 2016; Verschaeve et al. 2016). Prior to the adoption of the 2030 Agenda, this included efforts to support integrated target setting (see, e.g., Griggs et al. 2013, 2014; Nilsson et al. 2013). The policy coherence and institutional interaction literatures have often applied a “binary” view of interactions and focused on whether interaction is either beneficial or adverse (see, e.g., Oberthür and Gehring 2006). Similarly, a focus on the existence of “trade-offs” and “synergies” has dominated the discourse in the SDG policy debate. While this research has been helpful to establish that links exist, it does not provide sufficient information to guide policy. More recently, attempts have been made to establish a more nuanced way of viewing links and offer potential for a more elaborate understanding of interaction and the resulting policy implications. Weitz et al. (2014) applied three forms of interaction in their analysis of the water–energy–food nexus in the SDGs: interdependence, imposing conditions or constraints, and reinforcing (see also Coopman et al. 2016; UNESCO 2016). As a conceptual basis for a science-based assessment of interactions, Nilsson et al. (2016) and ICSU (2016) presented a seven-point typology of interaction, ranging from cancelling, counteracting, and constraining on the negative side to enabling, reinforcing, and indivisible on the positive side. ICSU (2017) applies this typology from the perspective of one SDG area.

To summarise, we note a gap in research about how SDG targets interact that (i) treats the 2030 Agenda as a whole (i.e., does not take one specific policy area, goal or target as the entry point and analytical focus, but considers their role within the system given how all targets interact) and (ii) considers how target interactions would play out in a given context with its specific geography, governance arrangements, and technological options.

In the following section, we briefly describe our analytical approach and methods. For ease of reading, the methodological application is presented along with the results in a sequence of steps in “Results”. In “Discussion of findings”, we discuss the results and their policy implication. “Conclusion” concludes.

## Analytical approach and methods

First, we construct a simple *cross*-*impact matrix* to organize and aggregate knowledge about interactions between SDG targets for the case of Sweden. Since targets’ affect on one another is highly contextual (Nilsson et al. 2016), we argue that it is almost invariably necessary to situate the assessment in a particular context. This consideration is based on previous work (Weitz et al. 2015) which stressed the need to interpret the target for a specific setting—with respect to the current status and trends on the issues that a target raises in that setting, the given resource base and policy efforts, as well as the objective stated in the 2030 Agenda that each government will set its own targets taking into account national circumstances (UN 2015).

In this paper, Sweden was selected because of the research team’s familiarity with the context (see, e.g., Weitz et al. 2015) as well as good data availability and chances to verify results with relevant stakeholders, which enabled relatively swift scoring. In addition, Sweden is an interesting case due to its government’s high ambition to be a front runner on SDG implementation and also the government’s interest in the policy coherence agenda (O’Connor et al. 2016).

The analysis is done at the level of targets, not at goal level, because targets are much more specific and this is where the substantive interactions are more easily discerned (ICSU 2017). Two targets per goal were selected, i.e., a total of 34 targets, see Table 1. This renders a total of 1122 interactions to be analyzed (34 × 33). The selection was based on a consideration of what are the most relevant and salient targets for each SDG in the context of Sweden (and excluding the “means of implementation-targets”).

**Table 1:** The 34 targets with official descriptions selected for Sweden

Target	Short description	Official description
1.3	Social protection	Implement nationally appropriate social protection systems and measures for all, including floors, and by 2030 achieve substantial coverage of the poor and the vulnerable
1.5	Economic and social resilience	By 2030, build the resilience of the poor and those in vulnerable situations and reduce their exposure and vulnerability to climate-related extreme events and other economic, social and environmental shocks and disasters
2.2	Malnutrition	By 2030, end all forms of malnutrition, including achieving, by 2025, the internationally agreed targets on stunting and wasting in children under 5 years of age, and address the nutritional needs of adolescent girls, pregnant and lactating women and older persons
2.4	Food production/ agriculture	By 2030, ensure sustainable food production systems and implement resilient agricultural practices that increase productivity and production, that help maintain ecosystems, that strengthen capacity for adaptation to climate change, extreme weather, drought, flooding and other disasters and that progressively improve land and soil quality
3.4	Non-communicable disease	By 2030, reduce by one-third premature mortality from non-communicable diseases through prevention and treatment and promote mental health and well-being
3.8	Health coverage	Achieve universal health coverage, including financial risk protection, access to quality essential health-care services and access to safe, effective, quality and affordable essential medicines and vaccines for all
4.1	Primary and secondary education	By 2030, ensure that all girls and boys complete free, equitable and quality primary and secondary education leading to relevant and effective learning outcomes
4.4	Technical/vocational skills	By 2030, substantially increase the number of youth and adults who have relevant skills, including technical and vocational skills, for employment, decent jobs and entrepreneurship
5.4	Unpaid/domestic work	Recognize and value unpaid care and domestic work through the provision of public services, infrastructure and social protection policies and the promotion of shared responsibility within the household and the family as nationally appropriate
5.5	Women’s participation	Ensure women’s full and effective participation and equal opportunities for leadership at all levels of decision-making in political, economic and public life
6.5	Water resources management	By 2030, implement integrated water resources management at all levels, including through transboundary cooperation as appropriate
6.6	Water-related ecosystems	By 2020, protect and restore water-related ecosystems, including mountains, forests, wetlands, rivers, aquifers and lakes
7.2	Renewable energy	By 2030, increase substantially the share of renewable energy in the global energy mix
7.3	Energy efficiency	By 2030, double the global rate of improvement in energy efficiency
8.4	Resource efficiency	Improve progressively, through 2030, global resource efficiency in consumption and production and endeavour to decouple economic growth from environmental degradation, in accordance with the 10-year framework of programmes on sustainable consumption and production, with developed countries taking the lead
8.5	Employment	By 2030, achieve full and productive employment and decent work for all women and men, including for young people and persons with disabilities, and equal pay for work of equal value
9.4	Infrastructure	By 2030, upgrade infrastructure and retrofit industries to make them sustainable, with increased resource-use efficiency and greater adoption of clean and environmentally sound technologies and industrial processes, with all countries taking action in accordance with their respective capabilities
9.5	Research/development	Enhance scientific research, upgrade the technological capabilities of industrial sectors in all countries, in particular developing countries, including, by 2030, encouraging innovation and substantially increasing the number of research and development workers per 1 million people and public and private research and development spending
10.1	Economic equality	By 2030, progressively achieve and sustain income growth of the bottom 40% of the population at a rate higher than the national average
10.7	Migration	Facilitate orderly, safe, regular and responsible migration and mobility of people, including through the implementation of planned and well-managed migration policies
11.1	Affordable housing	By 2030, ensure access for all to adequate, safe and affordable housing and basic services and upgrade slums
11.2	Transport	By 2030, provide access to safe, affordable, accessible and sustainable transport systems for all, improving road safety, notably by expanding public transport, with special attention to the needs of those in vulnerable situations, women, children, persons with disabilities and older persons
12.1	Sustainable consumption/production	Implement the 10-year Framework of Programmes on Sustainable Consumption and Production Patterns, all countries taking action, with developed countries taking the lead, taking into account the development and capabilities of developing countries
12.5	Waste	By 2030, substantially reduce waste generation through prevention, reduction, recycling and reuse
13.1	Climate change adaptation	Strengthen resilience and adaptive capacity to climate-related hazards and natural disasters in all countries
13.2	Climate change policy/planning	Integrate climate change measures into national policies, strategies and planning
14.1	Marine pollution	By 2025, prevent and significantly reduce marine pollution of all kinds, in particular from land-based activities, including marine debris and nutrient pollution
14.4	Fishery	By 2020, effectively regulate harvesting and end overfishing, illegal, unreported and unregulated fishing and destructive fishing practices and implement science-based management plans, to restore fish stocks in the shortest time feasible, at least to levels that can produce maximum sustainable yield as determined by their biological characteristics
15.2	Forests	By 2020, promote the implementation of sustainable management of all types of forests, halt deforestation, restore degraded forests and substantially increase afforestation and reforestation globally
15.5	Biodiversity	Take urgent and significant action to reduce the degradation of natural habitats, halt the loss of biodiversity and, by 2020, protect and prevent the extinction of threatened species
16.4	Illicit financial/arms flow	By 2030, significantly reduce illicit financial and arms flows, strengthen the recovery and return of stolen assets and combat all forms of organized crime
16.6	Effective institutions	Develop effective, accountable and transparent institutions at all levels
17.11	Exports from developing countries	Significantly increase the exports of developing countries, in particular with a view to doubling the least developed countries’ share of global exports by 2020
17.13	Macroeconomic stability	Enhance global macroeconomic stability, including through policy coordination and policy coherence

A larger or smaller selection of targets can be selected, depending on available resources and what is seen as relevant for a specific actor (such as an industry, government, or municipality) in a specific place. However, covering all 169 targets and how they interact is probably not feasible.

**Fig. 1:**
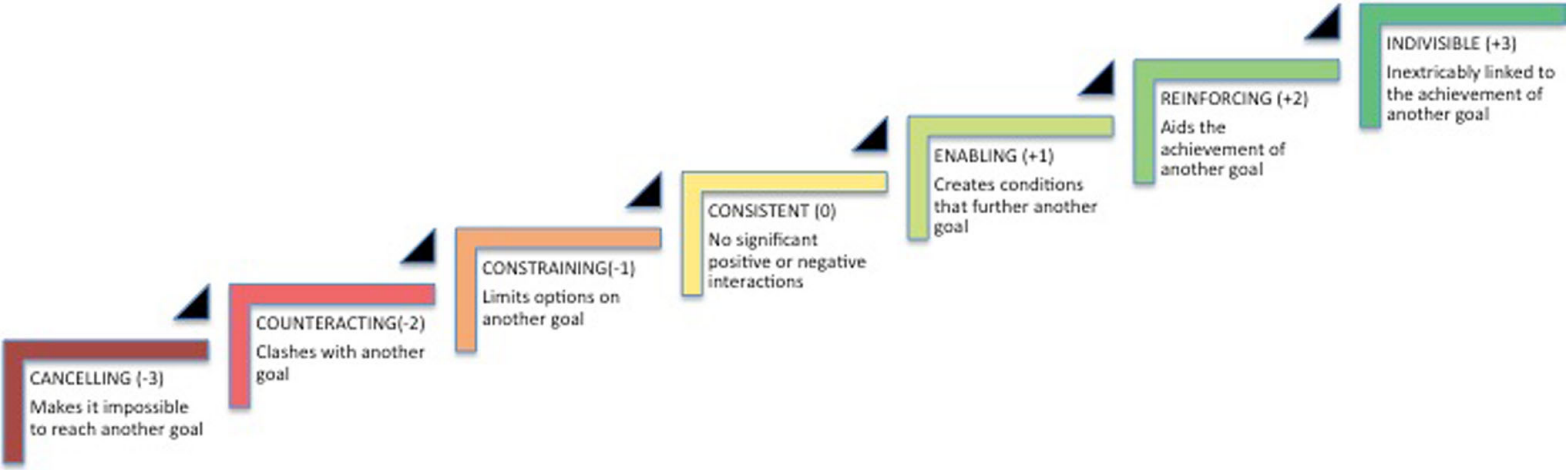
Seven-point typology of SDG interactions (Adapted from Nilsson et al. (2016))

To determine the interaction score, we used a recent conceptualization of interactions between policy areas (here SDGs), moving beyond the common but overly simplified dichotomy of synergies vs. trade-offs. The conceptualization was first presented in Nilsson et al. (2016), and has recently been applied in a more qualitative way from the perspective of individual SDGs in ICSU (2017). Nilsson et al. (2016) proposed a seven-point typology of the nature of interactions ranging from cancelling (−3), counteracting (−2), and constraining (−1) on the negative side, consistent (0) when there is no significant interaction, to enabling (+1), reinforcing (+2), and indivisible (+3) on the positive side, as shown in Fig. 1.

We use this typology and associated scoring to fill in the cross-impact matrix for the 34 selected targets. The cross-impact matrix is a tool designed for analyzing relationships between variables, factors, events, etc. The cross-impact matrix is the central database in a group of system analysis methodologies under the heading of cross-impact analysis (Gordon and Hayward 1968). The matrix elements of the cross-impact matrix contain numbers which describe how the occurrence of the row variable would affect the column variable. Most often, expert judgements are used for collecting the data.

Using the cross-impact matrix as the basis for analysis allows us to maintain a comprehensive view of the 2030 Agenda. In other words, we do not pre-select a certain area as the focus or entry point, but assess how all 34 targets interact with each other. From there, we can still draw sector-specific insights. This approach is in contrast to most previous attempts at looking at interactions, which explore one-on-one interactions from one sector to others and do not account for systemic effects (see, e.g., Vladimirova and Le Blanc 2016; UNESCO 2016; Coopman et al. 2016; ICSU 2017). This paper represents the first application of the scale focusing on the quantitative scoring and for the “whole-of-government”, i.e., including all policy areas entailed in the SDGs.

Distributing the workload evenly, we divided the 1122 interactions between the four co-authors, who each made an expert judgment of the scoring of the interaction based on a basic reading of the literature and prior knowledge. Each score that was not straight-forward was referenced with an explanatory note. All scores were then cross-checked in a session with all authors present and if necessary adjusted based on the discussion. The scoring process here remains a qualitative and judgment-based exercise that can be made more robust through a variety of strategies, depending on purpose and what resources are available for the assessment.

While some patterns (e.g., the relative frequency of positive and negative interactions) are possible to discern directly from the cross-impact matrix, the ability to identify systemic properties of interaction calls for application of more sophisticated methodologies to analyse the information it contains. To this end, this paper utilizes tools and techniques from *network theory*.

Intuitively, a network is a structure consisting of a set of nodes (also called vertices) and a set of links (also called edges) connecting some of the nodes more formally, a network is an ordered pair *G* = (*N*, *L*) comprising a set of nodes (*N*) and a set of links (*L*), where *L* is a subset of the Cartesian product of *N*, i.e., *N* × *N.* If *N* = {*a,b,c*}, then *N* x *N* = {{*a,b*},{*a,c*},{*b,c*}} and one possible network is *G* = {*N* = {*a,b,c*}, *L* = {{*a,b*},{*b,c*}}}. This is an example of a simple network with three nodes *a, b,* and *c*, where *a* is connected to *b* and *b* is connected to *c*, but *a* is not connected to *c*. Additional features can be added such as directions of links (e.g., in a social network, if Adam knows who Bob is but Bob does not know who Adam is, then there is a directed link from Adam to Bob), weights of links (e.g., in a trade network, the link representing German export to the US is a link with higher weight than the link representing German export to Finland), and more than one link between nodes (e.g., in a network of airports, there are many airline companies flying between Heathrow and Schiphol). Recently, networks have become useful for representing and analyzing a wide variety of systems, including biological (e.g., food webs), social (acquaintances), technological (internet), and information networks (www).

Network theory provides a wide range of tools and techniques for analyzing networks (for a comprehensive review of network theory including applications, see, e.g. Newman 2010). Network theory allows us to visualize the network of interactions and assess that the role targets play within the whole system.

In the following section, we guide the reader through the semi-quantitative analysis of interactions amongst all 34 targets applying this systemic and contextual approach.

## Results

Our results are presented below in a number of steps. First, some observations based on the cross-impact matrix are presented, including the frequency of positive and negative links, which targets are the most connected, and rankings of which targets that on balance are the most and least influenced and influencing ones. Second, all the links within the network, with their strength and direction, are visualized, and we look further into the most positive and most negative interactions. We also exemplify the influence from and on other targets from the perspective of one specific target. Third, we proceed to considering how interactions ripple through the system by looking at second-order interactions. Here, we show how considering systemic impacts alters the ranking of which targets can be considered the most influencing and influenced, and thus, how action may be reprioritized. Finally, we present how targets form clusters that reinforce each other and have shared interests that encourage collaboration amongst different actors.

### Cross-impact matrix

The analysis starts from the cross-impact matrix of interactions between the 34 targets. The scoring was guided by the question: *“If progress is made on target x (rows), how does this influence progress on target y (columns)”?* We were thus interested in the interaction that occurs when making progress on a target to another target, and not the interaction that would emerge from fully achieving it.

Each characterization and scoring in principle merits a substantial discussion and validation, which is not possible to account for in this paper. However, to give the reader an idea of the reasoning, we give two examples:

- Target 4.1 (*primary and secondary education*) was interpreted with emphasis on quality schooling for all children, which relates to a highly salient on-going political debate about the school system in Sweden. This target only has positive or neutral interactions with other targets. For example, equal education enables the poor to get coverage by *social protection* systems (target 1.3) and reinforces *resilience against economic and social change* (target 1.5) (Weitz et al. 2015).
- Target 6.5 (*water resources management*) displays mostly positive interactions but also two constraints with other targets. For example, it places constraints on the ways in which *renewable energy* can be expanded (target 7.2). The reason is the importance of hydropower in the Swedish energy system providing balancing capacity for the expansion of variable wind and solar power. This trade-off between water protection policy and hydropower is well-researched and subject to intense political debate.

**Fig. 2:**
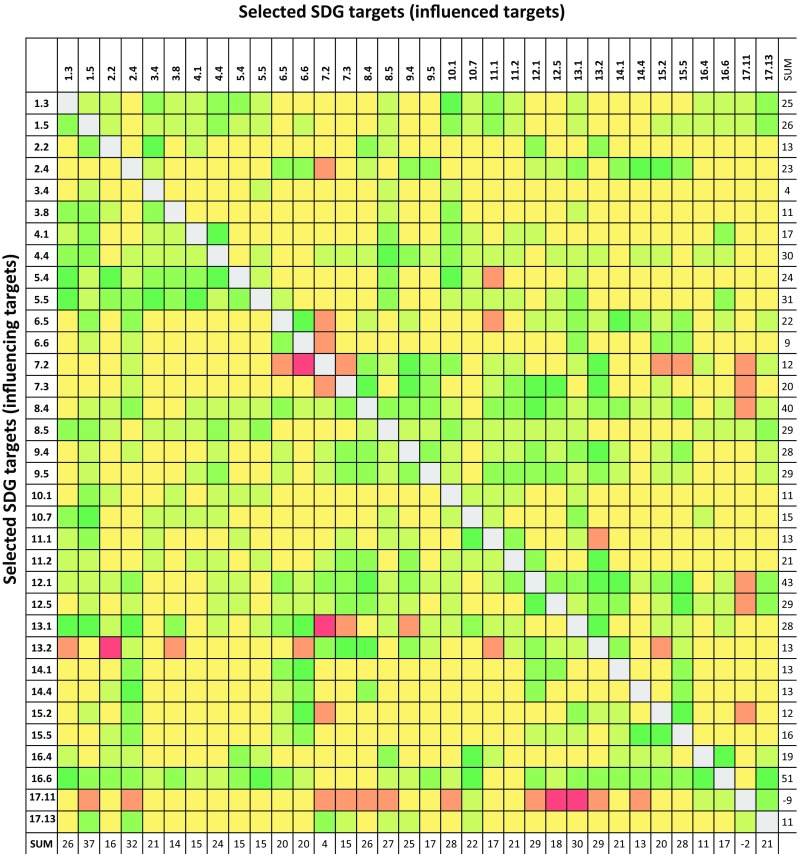
Cross-impact matrix of 34 targets and their interaction in Sweden. *Colour* according to scale in Fig. 1: from *dark red* (−3/cancelling) to *dark green* (+3/indivisible). The net influence from a target on all other targets is shown by the row-sum and the column-sum shows how much a target is influenced by all other targets in total

The cross-impact matrix resulting from analysis of all 1122 interactions is shown in Fig. 2. This matrix can be examined from different perspectives, and it provides the input data for all subsequent analytical steps.

At first sight, it is noteworthy that the interaction scale is seriously tipped to the positive-neutral side (of the total number of links, only 4% are red); that is, the targets generally exert more positive than negative influence on each other and making progress in one area makes it easier for other targets to be achieved. However, there are important exemptions as indicated by the red matrix elements, as shown in Fig. 2. The targets having the highest count of negative interactions with other targets are targets 17.11 (*exports from developing countries*, count 18), 7.2 (*renewable energy*, count 13), and 13.2 (*climate change policy and planning*, count 8).

The net influence accounts for whether the interaction is positive or negative and to what degree on the seven-point scale. The net influence from a target on all other targets is shown by the row-sum, as shown in Fig. 2, whereas the column-sum shows how much a target is influenced by all other targets in total.

A high row-sum suggests that a target has a large net positive influence on other targets. On balance, a target with a high row-sum can be seen as a synergistic one that makes the realization of other targets easier. A negative row-sum suggests that progress on that target generally makes it more difficult to achieve other targets. However, the row-sum does not show if that influence consists of a large number of weak influences on many targets or a few really strong ones, nor the distribution between positive and negative links. Target 16.6 (*effective institutions*) has the highest row-sum (sum 51); that is, it is the most positively influencing target and it exerts only positive influence (no red matrix elements). Target 12.1 ranks second (sum 43) and 8.4 third (sum 40), but despite their high rank as positive influencers, they also exert negative influence on some targets (−1 in both cases). The lowest row-sum is held by target 17.11 (sum −9), which thus exerts the least positive net influence on other targets. In fact, it exerts a negative influence of −14. Target 3.4 ranks second (sum 4) exerting only positive influence, whereas target 6.6 (*water*-*related ecosystems*) ranks third (sum 9) and exerts a negative influence of −1.

A high column-sum suggests that a target is greatly positively influenced by other targets. A negative column-sum means that progress in other targets makes it more difficult to reach the target. Progress on these highly influenced targets is reliant on developments in other targets and their independence or control over their own progress is low. Again, the sum does not show whether influence results from strong influence by a few targets or weak influence by many, or the distribution between positive and negative links. Target 1.5 has the highest column-sum (sum 37); that is, it is the most positively influenced target, but it also receives negative influence (−1). Target 2.4 (*food production/agriculture*) ranks the second most positively influenced target (sum 32) and also receives negative influence (−1), as well as target 13.1 which ranks third (sum 30) and receives a negative influence of −2. Target 17.11 has the lowest column-sum (sum −2); that is, it is the least positively influenced target. It yet receives a positive influence of 4 and negative influence of −6. Target 7.2 ranks second (sum 4), but receives even more negative influence (−8). Target 16.4 (*illicit financial/arms flow*) is the third least positively influenced target (sum 11), but receives no negative influence from other targets.

From the cross-impact matrix, we further note that only one target (17.11) has a negative column and row sum. This result, that increased exports from developing countries would make it more difficult to achieve the SDGs in Sweden (and that making progress on SDGs in Sweden would make it more difficult to achieve target 17.11), can be explained by the fact that potential benefits from trade are poorly captured in the 2030 Agenda. Benefits would include, for example, affordable consumer prices or greater variety of goods. We, however, note that positive interaction with *affordable housing* (target 11.1) has been captured.

The summing up of rows and columns provides an overview of targets’ net influence on other targets and whether they are strongly influenced by progress in other targets, but it does not provide enough information to guide priority-setting of where to focus action. To serve that purpose, we need more nuanced information about how the interaction between targets relates to the rest of the network, where strong positive and negative links are positioned.

### Applying network theory for analysis and visualization

To advance the analysis and also to visualize results, we now turn to network analysis techniques to interpret our data.

From a network perspective, the cross-impact matrix provides rather complex information, which makes analysis and visualization complicated. It does not only show that targets are linked but also includes the direction of links (i.e., influence points to or from targets), their weight (i.e., the strength of influence vary), and how they are “signed” (i.e., links can be either negative, neutral, or positive). In addition, the network is a multigraph; that is, it allows more than one link between a pair of targets, since their interaction is not symmetric.

**Fig. 3:**
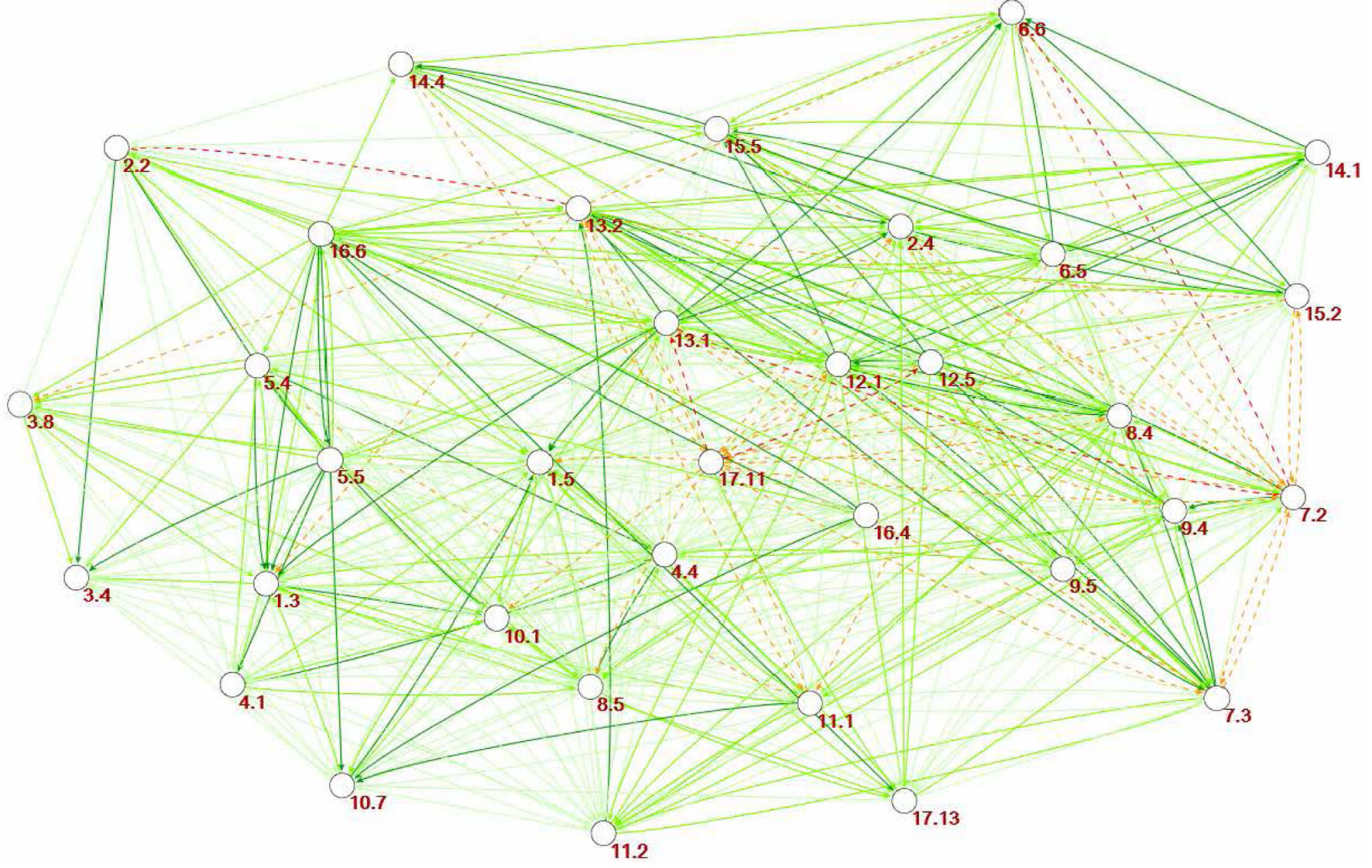
Full network: links between the 34 targets based on the cross-impact matrix in Fig. 2. *Colour scale* as in Figs. 1 and 2 and negative links *dashed*. *Arrows* show the direction of influence

As a first step, we translate the cross-impact matrix in Fig. 2 to a network depicting the links between all targets, their direction, and strength, as shown in Fig. 3.^2^ Each target is a node and two targets are connected if the corresponding matrix element in the cross-impact matrix is of any colour but yellow (which represent no significant interaction): Green links represent positive interactions and red and orange dashed links represent negative interactions. This is a visual representation of the information in the cross-impact matrix and the network does not contain any additional information compared to the cross-impact matrix. Compared to the cross-impact matrix, this network visualization is better in communicating the complexity of the problem at hand, and it is obvious that we need to go beyond simple network visualization and using tools from network theory to further analyse this structure.

**Fig. 4:**
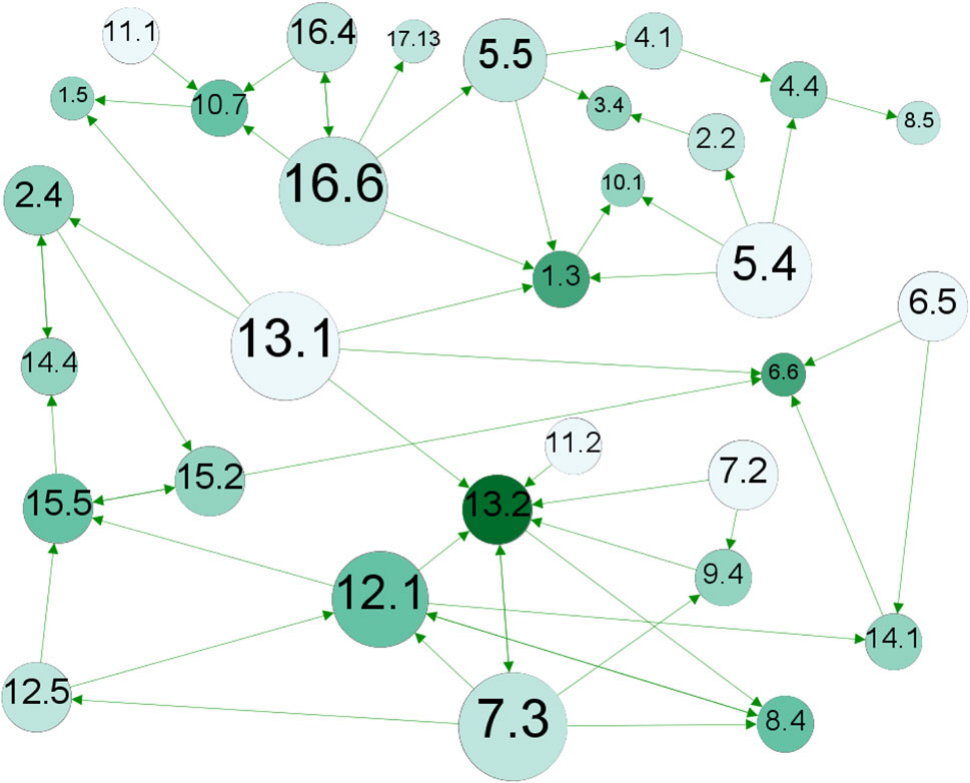
Sub-network of indivisible (+3) interactions. Directed as shown by *arrows*. The size of the nodes (targets) is proportional to the degree of influence (out-degree) with bigger nodes representing more influential nodes. The *colour* is proportional to the degree of being influenced with *darker colour* for nodes more influenced by other nodes

With the comprehensive underlying information base, we can further extract bits and pieces of information that are of particular interest for policy-making, exemplified by the network of only the most positive (+3) interactions, as shown in Fig. 4.

This sub-network nuances the picture of the sum of influence, which did not reveal the composition of negative and positive links from the cross-impact matrix. In the network, bigger nodes are more influential than smaller nodes and darker nodes are more influenced than clearer nodes. Targets 7.3 (*energy efficiency*), 13.1 (*Climate change adaptation*), and 16.6 (*Effective institutions*) are shown to exert strong positive influence on other targets, both with 5 outgoing +3-links. Targets 5.5 (*women’s participation*) and 5.4 (*unpaid/domestic work*) also exert strong positive influence. Comparing the initial analysis of most influencing targets, which was based on the sum of influence, 16.6 and 5.5 show to be strong influencers in both lists, whereas 5.4 was not initially ranked top five. Targets 12.1 (*sustainable consumption/production*), 8.4 (*employment*), and 4.4 (*technical/vocational skills*) ranked as top-five net influencers, but score low in exerted strong (+3) positive influence.

Conversely, target 13.2 (*climate change policy/planning*) stand out as receiving the most positive influence with 6 incoming +3 links. This is followed by targets 6.6 (*water*-*related ecosystems*) and 1.3 (*social protection*), and targets 15.5 (*biodiversity*), 10.7 (*migration*), and 8.4 (*employment*). Comparing again with the previous analysis only 15.5 matches. Targets 1.5 (*economic and social resilience*), 2.4 (*food production/agriculture*), 10.1 (*economic equality*), and 12.1 were ranked top-five most influenced, but receive few or none +3 links.

Target 13.2 (*climate change policy/planning*) is also the strongest positively connected target if we consider both in- and outgoing links. It has a total of 8 in- or outgoing +3 links; it benefits strongly from progress in targets 11.2 (*transport*), 7.2 (*renewable energy*), 9.4 (*infrastructure*), 7.3 (*energy efficiency*), 12.1 (*sustainable consumption/production*), and 13.1 (*climate change adaptation*), and strongly supports progress in targets 7.3 and 8.4 (*employment*). It can further be noted at this level of analysis that several targets—but particularly 5.4 (*unpaid/domestic work*) and 13.1 (*climate change adaptation*)—exert positive influence on several other targets without receiving positive influence back. We also note that only three targets 17.11 (*export from developing countries*), 9.5 (*research/development*), and 3.8 (*health coverage*) are not part of the sub-network of indivisible targets; that is, they neither receive nor exert such strong positive influence.

The analysis of indivisible links between targets suggests that investments in target 13.1 (*climate change adaptation*), 7.3 (*energy efficiency*), 16.6 (*effective institutions*), 5.5 (*woman’s participation*), and 5.4 (*unpaid/domestic work*) will generate additional progress, whereas progress in targets 13.2 (*climate change policy/planning*), 6.6 (*water*-*related ecosystems*), and 1.3 (*social protection*) is likely to be “automatic” as a result of progress in other areas. Attention can be directed to targets that have constraining or counteracting relationships with other targets, or those that overall receive little support from the network.

**Fig. 5:**
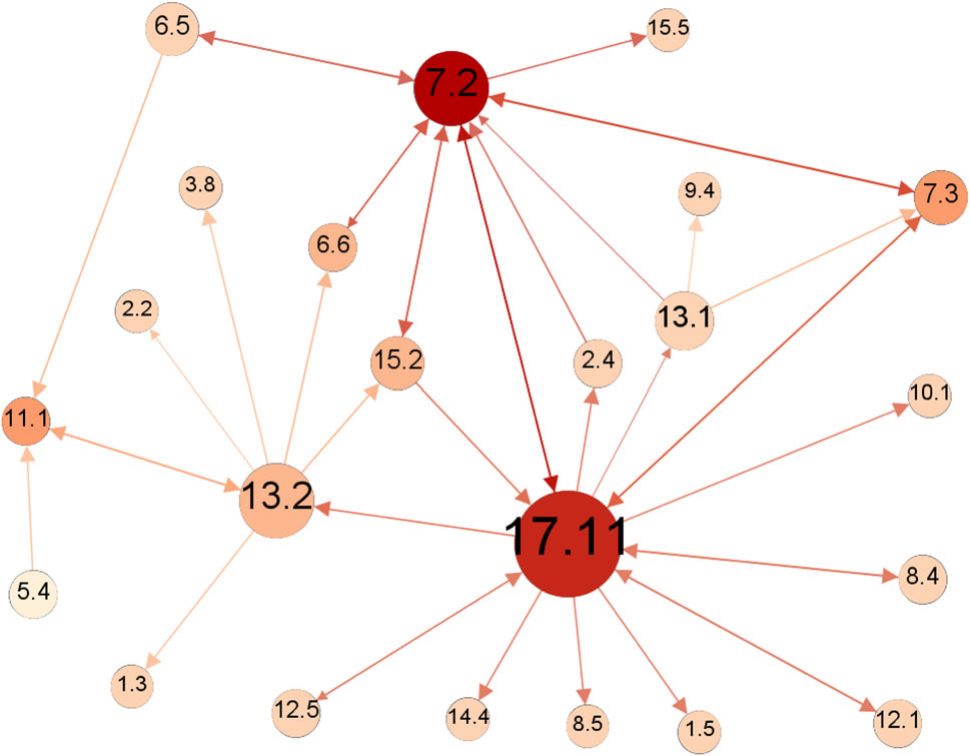
Sub-network of constraining (−1) and counteracting (−2) interactions. *Arrows* show the direction of influence. The larger the size of the node the greater the influence on other targets, and the darker the shade of the node the more influenced by other targets. Where multiple links exist between two nodes, they have been combined in *one arrow*, and their combined strength is indicated by the *shade of the arrow*: *dark red* (−2, −2 = −4), *red* (−1, −2 or −2, −1 = −3), *orange* (−1, −1, or −2 = −2), and *peach* (−1)

The sub-network of negative interactions is shown in Fig. 5 (in which both constraining (−1) and counteracting (−2) links are shown; there was no cancelling (−3) links).

In Fig. 5, target 17.11 (*export from developing countries*) stands out as being both highly negatively influenced by other targets and having a strong negative influence on other targets. Target 7.2 (*renewable energy*) shows the same pattern, but exerts less negative influence on other targets. Target 13.2 (*climate change policy/planning*) has as strong a negative influence on other targets as 7.2, but is less negatively influenced. It can also be noted that many targets (32%) do not feature in the graph; this is because they do not have any negative interactions, neither in- nor outgoing.

**Fig. 6:**
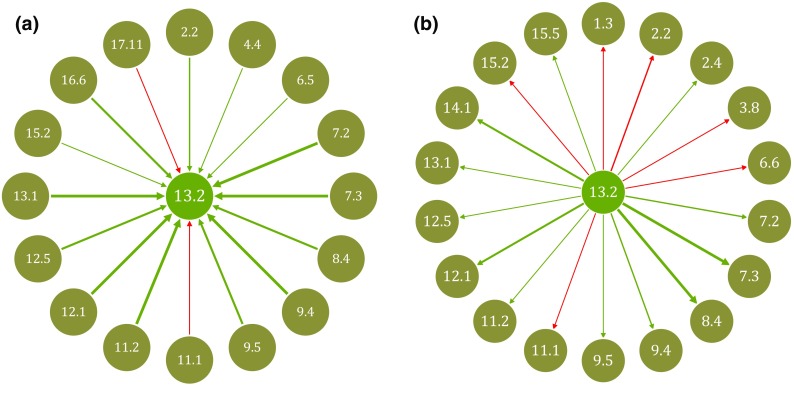
**a** Network from the perspective of target 13.2: influence from other targets. The *thicker the arrow* the stronger the influence from another target (−3 to +3). Negative influence in *red*, positive in *green*. **b** Network from the perspective of target 13.2: influence on other targets. The *thicker the arrow* the stronger the influence on other targets (−3 to +3). Negative influence in *red*, positive in *green*

From a policy-maker’s perspective, it might also be of interest to return to a partial policy perspective and visualize the influence from and on other targets from the perspective of one target. Consider, for example, a ministry in charge of climate mitigation measures [built into target 13.2 (*climate change policy/planning*)]. This ministry would first of all like to know which other targets affect its ability to progress on target 13.2 and to find out on whom it depends and would need to foster good collaboration with. This is shown in Fig. 6a. Second, the ministry will also be interested in understanding how its targets are affecting others, where it might meet resistance or will need to negotiate. This is shown in Fig. 6b. Target 13.2 directly influences 18 of the 33 other targets and is directly influenced by 16 of them.

This analysis only takes into account the interaction between one selected target [13.2 (*climate change policy/planning*)] and its immediately “neighbouring” targets, and does not consider how that neighbouring target in turn interacts with other targets. Hence, we have only included first-order interactions. This is useful information, but does not give us confidence about what areas should be prioritized to maximize progress overall. For example, as shown in Fig. 6a, progress on four of the top-five net influencers [16.6 (*effective institutions*), 12.1 (*sustainable consumption/production*), 8.4 (*resource efficiency*), and 4.4 (*technical/vocational skills*)] give much support to target 13.2 but target 13.2 in turn has a negative influence on six other targets (1.3 (*social protection*), 2.2 (*malnutrition)*, 3.8 (*health coverage*), 6.6 (*water*-*related ecosystems*), 11.1 (*affordable housing*) and 15.2 (*forests*)), as shown in Fig. 6b. Progress in the four top-influencing targets would render achievement of six other targets more difficult to achieve and prioritizing them might prove a counterproductive strategy if we look to impacts deeper into the network. To avoid this, decision makers need to be guided by analysis that takes into account how a target sits in relation with other targets given how all targets interact; that is, we need to understand its systemic impact within the influence network.

#### Guiding prioritization of action

To generate information that can guide prioritization of action, and raise warning flags, where extra attention is warranted to avoid negative impact, we need to account for how influence ripple through the network. If a target reinforces another target, which in turn has many and/or strong positive connections, its systemic impact can be very significant. If the other target has few and/or weak positive connections, the positive effect, however, wears out quickly without having much systemic impact. In addition, many strong positive connections to other targets with the same characteristics give a high and positive multiplier effect. Conversely, a strong positive connection to a target that in turn exert much negative influence on other targets makes a negative systemic impact, and must be avoided. A negative connection to a target that in turn has strong positive connections may be a reason for caution as negative impact can spread.

To test the hypothesis that prioritization of targets will change if second-order effects are taken into account, we calculate the net influence on the second-order network for all 34 targets in our selection. That is, we include the influence of the neighbouring target’s neighbour.

**Fig. 7:**
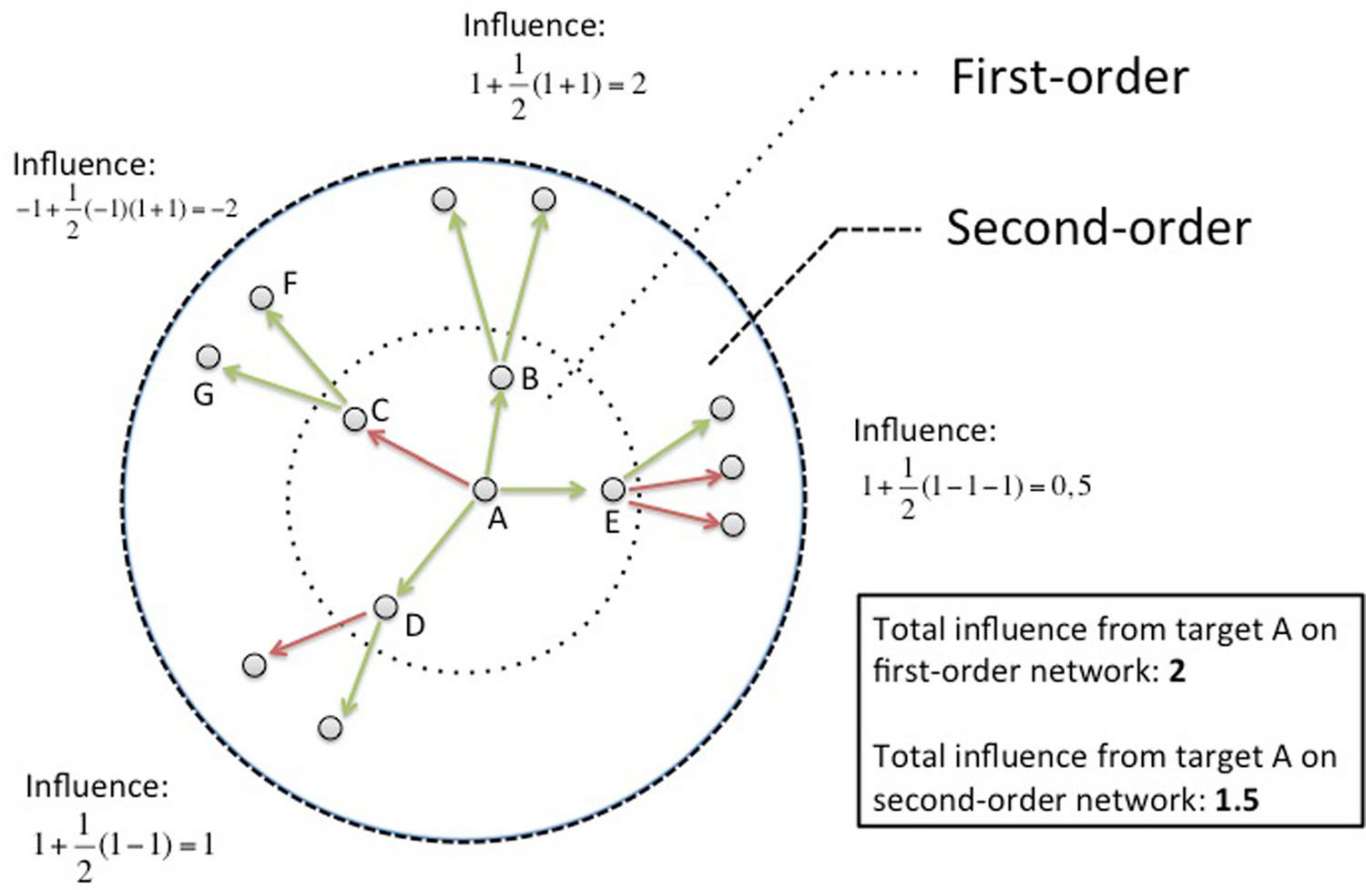
Conceptual figure showing the total influence by a target when considering first- and second-order interactions. For simplicity, the figure only includes +1 (*green arrows*) and −1 (*red arrows*) interactions. The approach is trivially generalized to the full scale [−3, −2, …, +3]. Calculating the total influence of *A* on the first-order network, we simply sum up the *arrows* in the *inner circle*: 3(+1) + 1(−1) = 2. Calculating the influence of A on second-order interactions, we consider the full chain of influence (e.g., from *A* to *F* and *G* via *C*). Here, *A*’s influence on *F* is not equal to the sum of the two links between *A* and *C* and *C* and *F* for two reasons: first, because the *A*–*C* link is negative, it makes progress in *C* more difficult and the positive influence that *C* would exert on *F* if progress was made less likely. Second, because influence weakens the further away from target *A,* it is exerted. Calculating *A*’s influence on *F,* we account for these effects by reducing the weight of the second-order links by 0.5 before multiplying the second-order links with the first-order link and adding this to the first-order influence. Adding up the total influence from the four chains of influence in the figure, the total influence from target *A* on the second-order network is 1.5

Figure 7 shows the conceptual idea of moving from only considering first-order interactions to second-order interactions. It shows how the net influence on the network generated by a target changes if we include second-order interactions in the assessment. Figure 7 highlights how a positive link in the second layer does not necessarily balance out a negative link in the first layer, but rather that the negative effect ripples and spread through the network.

The total influence (*I*) from target (*i*) on the second-order network is calculated as

$$I_{i}^{Total} = I_{i}^{1st} + \frac{1}{2}\mathop \sum \nolimits I^{2nd} = D_{i}^{Out} + \frac{1}{2} \mathop \sum \limits_{j \ne i} I_{ij} D_{j}^{Out}$$

where *I*_*i*_^*1st*^ is the influence of target *i* on its closest neighbours, *I* ^*2nd*^ is the influences from *i’s* neighbour’s on their neighbours weighted by a factor ½, *D*_*i*_^*Out*^ is the out-degree of target *i*, *I*_*ij*_ is the strengths of link from target *i* to target *j*, and *D*_*j*_^*Out*^ is the out-degree of target *j*. The result and rank of the targets are presented in Table 2 in parallel with the first-order ranking.

**Table 2:** Rank of positively influencing targets, first- and second-order networks

First-order network			Second-order network		
Rank	Target	Net influence	Rank	Target	Net influence
1	16.6 Effective institutions	51	1	16.6 Effective institutions	567
2	12.1 Sustainable consumption/production	43	2	12.1 Sustainable consumption/production	513
3	8.4 Resource efficiency	40	3	8.4 Resource efficiency	509
4	5.5 Women’s participation	31	4	12.5 Waste	381
5	4.4 Technical/vocational skills	30	5	9.5 Research/development	364.5
6	8.5 Employment	29	6	4.4 Technical/vocational skills	364
6	9.5 Research/development	29	7	5.5 Women’s participation	362.5
6	12.5 Waste	29	8	8.5 Employment	351
7	9.4 Infrastructure	28	9	9.4 Infrastructure	349.5
7	13.1 Climate change adaptation	28	10	7.3 Energy efficiency	322
8	1.5 Economic and social resilience	26	11	13.1 Climate change adaptation	312
9	1.3 Social protection	25	12	11.2 Transport	263.5
10	5.4 Unpaid/domestic work	24	13	6.5 Water resources management	262
11	2.4 Food production/agriculture	23	14	1.5 Economic and social resilience	261.5
12	6.5 Water resources management	22	15	5.4 Unpaid/domestic work	258.5
13	11.2 Transport	21	16	2.4 Food production/agriculture	257.5
14	7.3 Energy efficiency	20	17	1.3 Social protection	249.5
15	16.4 Illicit financial/arms flow	19	18	16.4 Illicit financial/arms flow	248
16	4.1 Primary and secondary education	17	19	4.1 Primary and secondary education	238.5
17	15.5 Biodiversity	16	20	13.2 Climate change policy/planning	224
18	10.7 Migration	15	21	7.2 Renewable energy	186
19	2.2 Malnutrition	13	22	10.7 Migration	174
19	11.1 Affordable housing	13	23	14.4 Fishery	173
19	13.2 Climate change policy/planning	13	24	2.2 Malnutrition	164
19	14.1 Marine pollution	13	25	14.1 Marine pollution	159.5
19	14.4 Fishery	13	25	15.5 Biodiversity	159.5
20	7.2 Renewable energy	12	26	11.1 Affordable housing	155
20	15.2 Forests	12	27	15.2 Forests	136
21	3.8 Health coverage	11	28	10.1 Economic equality	130
21	10.1 Economic equality	11	29	17.13 Macroeconomic stability	113.5
21	17.13 Macroeconomic stability	11	30	3.8 Health coverage	112
22	6.6 Water-related ecosystems	9	31	6.6 Water-related ecosystems	105.5
23	3.4 Non-communicable disease	4	32	3.4 Non-communicable disease	52.5
24	17.11 Exports from developing countries	−9	33	17.11 Exports from developing countries	−127.5

Comparing results for the first- and second-order network, the top-three ranking targets; that is, those exerting the greatest positive net influence on the network, are still 16.6 (*effective institutions*), 12.1 (*sustainable consumption/production*), and 8.4 (*resource efficiency*) for the second-order network, but then, 12.5 (*waste*) appears which previously ranked no. 6. Target 5.5 (*woman’s participation*) has dropped from fourth to sixth places. The bottom-three ranking targets also remain the same as in the first-order network: 17.11 (*exports from developing countries*), 3.4 (*non*-*communicable diseases*), and 6.6 (*water*-*related ecosystems*). Considering second-order interactions thus seems to make less of a difference for targets with very large or very little influence in the first-order network, whereas the effect is more pronounced for targets ranking somewhere in the middle. For example, target 1.3 (*social protection*) moves from the 9th to 17th places, 7.3 (*energy efficiency*) from 14th to 7th, and 15.5 (*biodiversity*) from 17th to 25th places. It should be noted here that the rank does not show the distribution of positive and negative links that make up a target’s influence, and a high-ranking target may still hold negative links that merit special attention by policy makers. Overlooking them would have negative implications for certain targets.

The second-order network does give better account for the systemic impact of targets than first-order interactions only, and is arguably a more complete information base for priority setting. It is important to stress that this conclusion is based on the prerequisite that all targets potentially influence all other targets and that these influences should be taken into account when prioritizing targets. At this stage, the motivation for this thesis can only be mathematical, since prioritization can only be judged *after* policy interventions; and this is further complicated by the fact that alternative policy options cannot—in principle—be compared.

Decision makers may still opt for other alternatives, for political or (short term) economic reasons, but they would need to make a strong case if at the same time calling for integrated and coherent policy for sustainable development.

The question arises how deep into the network and chain of influence the assessment should go; is it worthwhile to also account for third-order neighbours and beyond? The ideal would be to rank the targets according to its interactions with the complete network. The literature of network theory contains many such ‘centrality measures’ (see, e.g., Newman 2010). However, since we are dealing with a complicated network that has multiple links between targets and is signed and weighted, the application of different centrality measures as implemented in the standard software (such as Pajek, Gephi and Mathematica) is not straightforward, and should be subject to further research.

#### Guiding collaboration between actors

In many networks, the distribution of links is unevenly distributed; they form clusters (also called groups, modules, and communities) of high concentrations of links with low concentrations of links in-between the clusters.^3^ The identification of such clusters within the network of SDG targets can help decision makers to develop comprehensive implementation strategies and organize implementation beyond just a ranking of individual targets as described in the previous section. Targets forming a cluster make a good coalition; they influence each other positively; and they have a shared interest in handling the negative links to other clusters. The set of actors corresponding to the targets included in a cluster may be different from the present logic of how responsibility is divided (e.g., across ministries by policy area or topic) and what is now perceived as important collaborations given shared or conflicting interests. Exploring clusters can thus present an effective way to organize SDG implementation and build strategic partnerships.

There are several algorithms for detecting clusters (for reviews, see, e.g., Caldarelli and Vespignani 2007; Fortunato 2010; Newman 2004; Schaeffer 2007) and the quality of these algorithms is often assessed via a measure known as modularity (Newman and Girvan 2004). The intuition underlying modularity is that a good division of the nodes into different clusters is one in which there are fewer links between the clusters than what is statistically expected; hence, it is not enough that there are simply ‘few’ links. Relatively little attention has been paid to the problem of detecting clusters in signed networks (Esmailian and Jalili 2015), and only recently has this functionality started to be implemented into software packages for network analysis (e.g., Pajek). The idea when identifying clusters in signed networks is to find groups of targets with few internal negative links and instead position the negative links between the clusters.

**Fig. 8:**
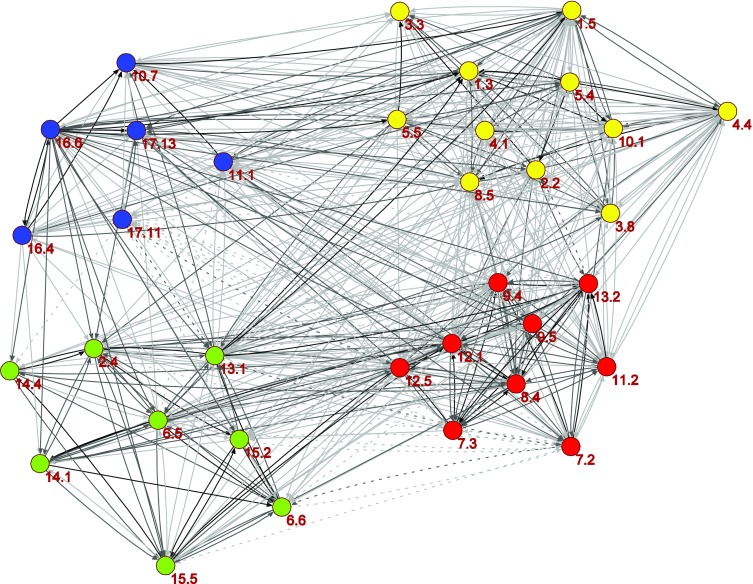
The 34 targets organized into four clusters. *Colour scale* from *light gray* to *black* with negative links *dashed*

Figure 8 shows the result of applying such a clustering algorithm to the full network. Only one negative interaction sits within one of the clusters (between targets 7.2 and 7.3 in the red cluster); all other negative interactions sit between targets that belong to different clusters.

A rapid examination of the clusters against how responsibility for the different policy areas covered by the SDG targets is divided across ministries; we find that a ministry’s area of responsibility or concern is often spread across clusters. For example, the blue and yellow clusters in Fig. 8 can be seen to broadly match the social sustainability ambitions of Swedish policies, whereas the green cluster matches issues related to environmental sustainability and the red cluster technology and innovation. While the ministries of Employment; Education and Research; Health and Social Affairs; Environment and Energy; and Enterprise and Innovation have overall responsibility for these policy areas, individual targets within the clusters crisscross current ministry lines, and an actor may both be a member of the yellow, red, green, and/or blue cluster. The clusters can thus provide guidance for an alternative way of dividing responsibility for SDG implementation; highlight the dual roles and the importance of a ministry’s involvement in many different constellations and policy areas; and provide ministries with an overview of how realizing their interests may require joining up with or establishing new collaborations that may at first seem distant.

## Discussion of findings

We approached a systemic and contextual analysis of SDG interactions with the hypothesis that giving consideration to systemic impacts would alter which efforts should be prioritized to enhance the effectiveness of implementation strategies. Our results have supported this. However, the impact appear least pronounced on targets ranking top- and bottom-three in terms of exerting positive influence on the network (shown in Table 2). Here, we discuss the implications of our findings for priority setting of actions and the organization of implementation.

A first, encouraging, finding is that the SDGs are dominantly mutually supportive in the case of Sweden; there are many more positive than negative links within the network and there is no instance of a target cancelling progress in another. Considering weighted second-order interaction, which is as deep as our analysis could go, we have found that progress in targets 16.6 (*effective institutions*), 12.1 (*sustainable consumption/production*), and 8.4 (*resource efficiency*) generate the most positive influence on the rest of the SDGs in Sweden. Making investments and generating progress here, positive knock on effects will ripple through the system. Being strong influencers, these targets drive progress on the 17 SDGs overall. Decision- and policy-makers with specific responsibility for and mandate to act on these target areas must realize how outcome hinges on their delivery, and work out how to best realize this potential in collaboration with other actors. Additional resources may also be channeled to these targets, not to diminish the importance of other policy areas, but just because they also benefit from this investment. The full network graph (Fig. 3) and analysis of how a certain target influences is being influenced by others [exemplified for target 13.2 (*climate change policy/planning*) in Fig. 6a, b] provide useful entry points for discussion amongst affected parties. Identifying how these top- and bottom-ranking targets cluster with other targets further provide an overview of potentially strategic partnerships that crisscross existing ministry lines.

When it comes to being influenced, targets 1.5 (*economic and social resilience)*, 2.4 (*food production/agriculture*), and 13.1 (*climate change adaptation*) receive the most positive influence from progress in other targets. Being supported by investments elsewhere, they may not need as much targeted support. However, progress in other targets cannot be assumed, and being influenced (whether positively or negatively) is rather an indication of high dependency on other targets. Progress in the most influenced targets is consequently more uncertain; control is distanced and unforeseen set-backs in other areas might spill over and postpone progress on the target in question, even if the measures aimed directly to the target are successful. Those with responsibility to implement a highly influenced target have the least control over their own issue area. Rather than surrendering to this fact, it should be a strong motivation to nurture relationships with the actors in charge of the targets that hold the key to their development. Because of their uncertainty, selecting a highly influenced target as a flagship target would not be very strategic, even if the potential influence is strongly positive. A number of targets receive very little support from other targets, including 17.11 (*exports from developing countries*), 7.2 (*renewable energy*), and 16.4 (*illicit financial/arms flows*), or are weakly connected to the rest of the network, including targets 14.4 (*fishery*), 14.1 (*marine pollution*), 3.4 (*non*-*communicable diseases*), and 3.8 (*health coverage*). Their dependence on progress in other targets is low and they have a lot of freedom to act independently. However, not benefitting from systemic effects, they may need more targeted support.

When we looked at the sum of positive and negative influences by and on a target (Fig. 2), we found only one target with an net negative influence: 17.11 (*exports from developing countries)*. Policies and measures to meet such targets must be handled with care as otherwise, other efforts can become neutralized. This is also true for any negative links that still exist within the generally positively reinforcing network, and stresses that it cannot be assumed that positive influence from one target evens out negative influence from another. The sub-networks of the strongest positive and negative links (Figs. 4, 5) brought useful information by raising warning flags and opportunities that should not be missed for some targets. It suggests that in addition to target 16.6 (*effective institutions*), which we have already concluded is likely to make a good investment, there are good reasons to ensure progress in target 13.1 (*climate change adaptation*) and 7.3 (*energy efficiency*) as that would exert strong positive influence on the network. Furthermore, we noticed that targets 13.2 (*climate change policy/planning*), 6.6 (*water*-*related ecosystems*), and 1.3 (*social protection*) receive a lot of support via other targets (but may also receive negative influence that counters this effect).

Conversely, 17.11 (*exports from developing countries*) and 7.2 (*renewable energy*) were found to both have a strong negative influence on, and be negatively influenced by, the network. 13.1 (*climate change adaptation*), 13.2 (*climate change policy/planning*), 11.1 (*affordable housing*), 7.3 (*energy efficiency*), and 6.5 (*water resource management*) are five other targets found to either exert much negative influence or be negatively influenced. These targets are potentially problematic and merit special attention, either because progress on them will make other targets more difficult to achieve or because achieving them will become more difficult if progress is made in other targets. The challenge posed by negative interactions depends on what the interaction entails and available means to mitigating it. Our findings indicate the strength of positive or negative interaction, but do not say anything about the challenge of undertaking the actions needed to capture or overcome that influence. This needs to be investigated on a case-by-case basis. Prioritizing progress in targets exerting negative influence on the network could still be a desirable strategy for political reasons or because negative influence can easily be overcome; they, however, raise red flags for policy makers to carefully consider.

The analysis of clusters provided interesting institutional insights. We identified four clusters of targets that are tightly positively connected (see Fig. 8). They match headline objectives of Swedish domestic and foreign policy including social and environmental sustainability, and technology and innovation, but responsibility for the policy areas of individual targets that make up the clusters belongs to different ministries. This suggests that bridges for collaboration are needed between ministries. The positive links between the targets that they are responsible for make an excellent entry point to foster this, as they highlight their shared interests and how they would benefit from progressing on targets.

A key message resulting from Fig. 6a, b, which looked at the network from one target’s perspective is that actors with responsibility to implement a target have many links and relationships to consider, even if only considering first-order interactions. It cannot be expected that they maintain the systemic overview in their daily work—that would be the responsibility of coordinating functions like the Prime Minister’s office—rather, they must internalize that it is in their interest to remind whoever influences them to explore who they in turn are influenced by and negotiate a way forward that generate the best possible outcome on the SDGs.

## Conclusion

We have demonstrated a practical approach for gaining a systemic and contextual perspective on the SDGs by building on network analysis and a simple typology of interactions. The approach is intuitively simple and can be applied in almost any country, regardless of data availability. Still, it is systemic in two ways: first by enabling a structured way to document and code interconnectedness between SDG targets and second by providing an approach for deriving system-wide effects from the initial understanding of interconnectedness.

The potential weakness of the approach is that the quality of the analysis depends on the scoring of interactions entered into the cross-impact matrix, so the approach is vulnerable to deficiencies in the scoring approach. If the matrix is not valid, the ensuing steps are pointless. The findings in this paper are only illustrative, as they rest on a test-run of the approach with limited in-house resources and where the scoring process remained a judgment-based exercise. This could be made more robust through a variety of strategies and depending on the purpose. For example, the data inputs could be reviewed by independent scientists or policy makers, or verified through systematic literature reviews. More specific methodologies to arrive at consensus amongst experts such as the Delphi method could be deployed (Blas et al. 2016). However, in our view, the preferred method for scoring is through a carefully facilitated workshop with policy makers and/or stakeholders. The policy dialogue and learning process that can then take place will be as important—or more—as the final results themselves. It also ensures better diversity of perspectives and political legitimacy of the outcome.

The key strength of the approach is not as a scientific assessment methodology but as a tool to support policy making, with a high degree of transparency and opportunity for engagement compared to modelling approaches. It induces sectors to look outside their turf and think systematically about how they influence, and are influenced, by others. It can also bring scientific knowledge into the policy-making process (using scientific data to underpin the scores) in a highly aggregated way which is suitable in a policy context. Everything can be easily traced back to original entries, and it is important that this transparency is maintained if other data collection methods are applied. Further transparency and diversity can be justified—for example, one could develop several variations of the cross-impact matrix if there are large uncertainties or disagreements about the score. How scoring variations play out systemically would be a useful sensitivity analysis.

Related to the scoring, the translation of information from qualitative to quantitative data opens up for a plethora of methods. Overall, the scale that we applied served this purpose well. However, our involvement in recent applications of the scale has shown that there can be a risk of confusing the scale as a typology coding the qualitative nature of an interaction with a scale of strength of interaction. The scale used here does not measure the strength of interaction (ICSU 2017).

We have already hinted at some possible extensions of the analysis. This paper has gone as far as to account for second-order interactions under the assumption that progress was made on a target. The method applied would also allow for exploring how lack of progress (or regression) on a target would influence other targets, assuming that this is not always symmetrical to the influence generated by progress. For example, in some areas, lack of progress may generate more negative impact than the positive impact generated by progress, or vice versa. A future research agenda could further include accounting for how target interactions ripple through the complete network, i.e., going beyond second-order interactions. This should also allow for identifying how targets might create positive feedbacks in virtuous and/or vicious circles.

Moreover, to fully support decision making that takes into account systemic impacts, a user-friendly interactive tool would be useful. The cross-impact matrix and network analysis lays the foundation for this.

This paper has responded to the expressed desire by the UN and several governments to treat the SDGs as an indivisible whole, the need for making SDG implementation a whole-of government endeavour and the importance of policy coherence for sustainability. We have pushed the frontier of how interactions amongst SDG targets can be understood and taken into account in SDG planning. We have stressed the importance of context-specific analysis of how interactions play out and exemplified how priority-setting changes if considering systemic impacts. The approach enables decision making that better accounts for how targets influence each other as part of a system, pointing to where policy intervention would be the most strategic to generate overall progress. The type of insights generated makes plans and priority setting for implementing the SDGs more likely to be effective.

## Electronic supplementary material

Below is the link to the electronic supplementary material.

Supplementary material 1 (DOCX 108 kb)

## Footnotes

1 A search in the Web of Science Core Collection for the topic “Sustainable development goals” and “Agenda 2030”, generated 642 articles (October 21st 2016). These have been screened and categorized.

2 This can be done using any freely available network visualization software package, e.g. Gephi (https://gephi.org), Pajek (http://mrvar.fdv.uni-lj.si/pajek/) or Netdraw (https://sites.google.com/site/netdrawsoftware/home).

3 There is no universally accepted quantitative definition of a cluster. Intuitively it is rather easy to think of a cluster as a collection of nodes in which the density of links is higher than the average density of the network. In reality, when actually identifying clusters, they are most often ‘algorithmically defined’, meaning that the set of clusters is simply the product of a certain algorithm without a precise definition (Fortunato 2010).
