# Supplementary material for: Towards systemic and contextual priority setting for implementing the 2030 Agenda
Source: Sustain Sci. 2017 Sep 12;13(2):531–48. doi: 10.1007/s11625-017-0470-0 (PMC6086277; doi:10.1007/s11625-017-0470-0)
Supplement: Supplementary file 1 — Supplementary material 1 (DOCX 108 kb) [file 11625_2017_470_MOESM1_ESM.docx]

Appendix 1. The 34 SDG targets selected for analysis

*As presented in Resolution* (A/RES/70/1) *adopted by the General Assembly on 25 September 2015 during the United Nations summit for the adoption of the post-2015 development agenda: Transforming our world: the 2030 Agenda for Sustainable Development.*

1. 1.3 Implement nationally appropriate social protection systems and measures for all, including floors, and by 2030 achieve substantial coverage of the poor and the vulnerable
2. 1.5 By 2030, build the resilience of the poor and those in vulnerable situations and reduce their exposure and vulnerability to climate-related extreme events and other economic, social and environmental shocks and disasters
3. 2.2 By 2030, end all forms of malnutrition, including achieving, by 2025, the internationally agreed targets on stunting and wasting in children under 5 years of age, and address the nutritional needs of adolescent girls, pregnant and lactating women and older persons
4. 2.4 By 2030, ensure sustainable food production systems and implement resilient agricultural practices that increase productivity and production, that help maintain ecosystems, that strengthen capacity for adaptation to climate change, extreme weather, drought, flooding and other disasters and that progressively improve land and soil quality
5. 3.4 By 2030, reduce by one third premature mortality from non-communicable diseases through prevention and treatment and promote mental health and wellbeing
6. 3.8 Achieve universal health coverage, including financial risk protection, access to quality essential health-care services and access to safe, effective, quality and affordable essential medicines and vaccines for all
7. 4.1 By 2030, ensure that all girls and boys complete free, equitable and quality primary and secondary education leading to relevant and effective learning outcomes
8. 4.4 By 2030, substantially increase the number of youth and adults who have relevant skills, including technical and vocational skills, for employment, decent jobs and entrepreneurship
9. 5.4 Recognize and value unpaid care and domestic work through the provision of public services, infrastructure and social protection policies and the promotion of shared responsibility within the household and the family as nationally appropriate
10. 5.5 Ensure women’s full and effective participation and equal opportunities for leadership at all levels of decision-making in political, economic and public life
11. 6.5 By 2030, implement integrated water resources management at all levels, including through transboundary cooperation as appropriate
12. 6.6 By 2020, protect and restore water-related ecosystems, including mountains, forests, wetlands, rivers, aquifers and lakes
13. 7.2 By 2030, increase substantially the share of renewable energy in the global energy mix
14. 7.3 By 2030, double the global rate of improvement in energy efficiency
15. 8.4 Improve progressively, through 2030, global resource efficiency in consumption and production and endeavour to decouple economic growth from environmental degradation, in accordance with the 10-Year Framework of Programmes on Sustainable Consumption and Production, with developed countries taking the lead
16. 8.5 By 2030, achieve full and productive employment and decent work for all women and men, including for young people and persons with disabilities, and equal pay for work of equal value
17. 9.4 By 2030, upgrade infrastructure and retrofit industries to make them sustainable, with increased resource-use efficiency and greater adoption of clean and environmentally sound technologies and industrial processes, with all countries taking action in accordance with their respective capabilities
18. 9.5 Enhance scientific research, upgrade the technological capabilities of industrial sectors in all countries, in particular developing countries, including, by 2030, encouraging innovation and substantially increasing the number of research and development workers per 1 million people and public and private research and development spending
19. 10.1 By 2030, progressively achieve and sustain income growth of the bottom 40 per cent of the population at a rate higher than the national average
20. 10.7 Facilitate orderly, safe, regular and responsible migration and mobility of people, including through the implementation of planned and well-managed migration policies
21. 11.1 By 2030, ensure access for all to adequate, safe and affordable housing and basic services and upgrade slums
22. 11.2 By 2030, provide access to safe, affordable, accessible and sustainable transport systems for all, improving road safety, notably by expanding public transport, with special attention to the needs of those in vulnerable situations, women, children, persons with disabilities and older persons
23. 12.1 Implement the 10-Year Framework of Programmes on Sustainable Consumption and Production Patterns, all countries taking action, with developed countries taking the lead, taking into account the development and capabilities of developing countries
24. 12.5 By 2030, substantially reduce waste generation through prevention, reduction, recycling and reuse
25. 13.1 Strengthen resilience and adaptive capacity to climate-related hazards and natural disasters in all countries
26. 13.2 Integrate climate change measures into national policies, strategies and planning
27. 14.1 By 2025, prevent and significantly reduce marine pollution of all kinds, in particular from land-based activities, including marine debris and nutrient pollution
28. 14.4 By 2020, effectively regulate harvesting and end overfishing, illegal, unreported and unregulated fishing and destructive fishing practices and implement science-based management plans, in order to restore fish stocks in the shortest time feasible, at least to levels that can produce maximum sustainable yield as determined by their biological characteristics
29. 15.2 By 2020, promote the implementation of sustainable management of all types of forests, halt deforestation, restore degraded forests and substantially increase afforestation and reforestation globally
30. 15.5 Take urgent and significant action to reduce the degradation of natural habitats, halt the loss of biodiversity and, by 2020, protect and prevent the extinction of threatened species
31. 16.4 By 2030, significantly reduce illicit financial and arms flows, strengthen the recovery and return of stolen assets and combat all forms of organized crime
32. 16.6 Develop effective, accountable and transparent institutions at all levels
33. 17.11 Significantly increase the exports of developing countries, in particular with a view to doubling the least developed countries’ share of global exports by 2020
34. 17.13 Enhance global macroeconomic stability, including through policy coordination and policy coherence
